# When the Past is Lost: Focal Retrograde Amnesia. Focus on the“Functional” Form

**DOI:** 10.3233/BEN-2008-0222

**Published:** 2009-07-29

**Authors:** Andrea Stracciari, Cristina Fonti, Maria Guarino

**Affiliations:** Neurology UnitS.Orsola-Malpighi University HospitalBolognaItaly

**Keywords:** Amnesia, focal retrograde amnesia, psychogenic amnesia, functional amnesia, transient global amnesia

## Abstract

We report the clinical findings and neuropsychological profiles of a sample of patients exhibiting a focal retrograde amnesia (FRA) seen consecutively during the period 1992–2007. The cohort comprised 13 patients, five males, with a mean age of 30 years (range 16–49). They were given a neurologic examination, psychiatric interview and formal neuropsychological examination (all but one) during the amnesic phase, underwent neuroimaging, and were followed up for six months to ten years. All presented with an acute amnesia characterized by an impaired recollection of memories predating the acute event, with spared or minimally and transiently affected anterograde memory, thus consistent with FRA. The events triggering FRA varied widely: mild to severe head injury, road accident without head injury, seizure, dissociative fugue, BDZ overdose, posttraumatic headache, syncope, migraine attack, acute distress. The neuropsychological hallmark of FRA was a selective or prominent impairment of autobiographical memory. The defect was often so severe as to cover most or all of the patients' lives and, in some cases, to erase the knowledge of their own identity. Conventional neuroimaging (brain CT and MRI) was unimpressive. Cerebral SPECT/PET disclosed unilateral frontal hypoperfusion in three (two left). All but one patient fully recovered, time of recovery ranging from three days to six months. FRA is a condition reflecting a block of memory function triggered by heterogeneous events, including both physical and psychic insults. Analogies shared with the more frequently encountered and better known condition of transient global amnesia suggests common pathogenetic mechanisms. A tentative nosographic classification of FRA is finally offered.

*"The past is a foreign country: they do things differently there"*

(*L.P. Hartley*)

## 1. Introduction

In recent years increasing interest has focused on patients exhibiting an acute memory dysfunction characterized by profound retrograde amnesia with minimal or absent anterograde amnesia, namely focal retrograde amnesia (FRA) [28,61,78]. Initially deemed a trivial psychiatric manifestation, hence scarcely investigated from a neurological point of view, FRA subsequently aroused the interest of neurologists and neuropsychologists, being observed associated with overt brain damage, such as severe head injury [32, 42], encephalitis (see [28,61,78] for a review), cerebrovascular insults [17,54] and acute cerebral hypoxia [48]. FRA has also been reported in association with a wide range of heterogeneous conditions such as seizures [34,47], trivial head trauma [14,46,70,73], cerebral headache/migraine [62,65,76] and drug intoxication [64], favoring the view of FRA as a syndrome [30,44]. The observation of FRA cases occurring in the course of acute emotional stress and also in the absence of clear causal or triggering factors, always associated with normal ancillary investigations, has led some to reconsider a psychogenic or 'functional' explanation – at least for such cases [5,6,38,39,51,52,68, 69]. This has kindled a heated debate on FRA concerning the organic/psychiatric dichotomy [1,3,11,29, 35]. To date, most authors have rejected the equation no lesions = psychogenic, and the term "functional", as

ISSN 0953-4180/08/$17.00 © 2008 – IOS Press and the authors. All rights reserved suggested by De Renzi and coworkers [13–15], refers to a block in function [20,31,36,44,49–51,53,76], not necessarily psychogenic in nature (but see Kritchevsky et al. [39] and Brandt and Van Gorp [5] for a different opinion). For practical purposes, the clinical distinction between lesional and non lesional forms of FRA tends to be maintained, but cases of lesional origin (e.g. cerebral lesions) and of a functional nature (e.g. emotional trauma) often share common clinical features.

In the last 15 years, we have encountered 13 patients presenting with features consistent with FRA, all construed as functional forms according to De Renzi and coworkers [13–15]. Four patients have been described in detail elsewhere [73,76]. We report here the clinical and neuropsychological profiles of the whole sample, with two main aims:

1) to delineate the main characteristics of functional FRA
2) to propose a tentative taxonomy of various forms of FRA.

This is the first sufficiently large sample of FRA patients seen by the same group and evaluated in a standardized manner and may thus yield a better understanding of FRA phenomenology.

## 2. Methods and patients

Subjects included patients consecutively seen by us from 1992 to 2007 exhibiting a cognitive dysfunction consistent with FRA. They all were examined by the same neurologist (AS) and underwent general ancillary investigations, psychiatric consultation, conventional neuroimaging (CT/MRI) and standardized neuropsychological assessment (all but one) during the amnesic phase. Nine patients also underwent cerebral SPECT; one patient underwent cerebral PET. Neuropsychological testing consisted of tasks exploring general cognition and memory, described in detail elsewhere [73, 76]. In particular, *general cognition* was explored with Wisconsin Card Sorting Test [55], Word Fluency [7], Phrases Construction [7], Raven's Coloured Progressive Matrices [7], Attentional Matrices [71] and copy of the Rey's Complex Figure [2]. *Anterograde memory* testing included: digit, verbal and spatial spans [59, 71], Immediate Visual Memory [7], Rey's 15 words [7], Paired Associate Learning [57], Story Recall [71], Spatial supra-span learning [71], Delayed Reproduction of Rey's Complex Figure [2]. *Retrograde memory tasks for public knowledge* included the Famous Events Questionnaire (Q 80) [12], the Face Identification and Face Familiarity check tests [18], and two ad hoc questionnaires concerning current famous public events (e.g. terrorist attacks, wars, crimes, international political or sporting events) and famous people (name familiarity check, "who is who", "dead/alive") [73,76]. *Autobiographical memory* (*ABM*) was assessed by means of a cued recall test with Crovitz and Schiffman procedure [21], and with the Autobiographical Interview [4].

## 3. Results

The results derived from the data of 13 patients, five males, with a mean age of 30 years (range 16–49). The case histories are briefly summarized. A more detailed account of four patients (case 1, 2, 4, 5) has been previously offered [73,76].

### 3.1. Case histories

#### 3.1.1. Patient 1 [73]

On 3rd March 1992 this 20-year-old healthy man suffered a mild head trauma during a minor car accident, with temporary global amnesia followed by a retrograde autobiographical amnesia for the last 12 months, involving both the retrieval of personally relevant events and the knowledge of personal important semantic memories (e.g. he was not aware of being in love with a girl, who seemed familiar but not so intimally related to him). He had normal general and neurological examination, blood parameters, ECG, EEG, brain CT and MRI. Cerebral SPECT disclosed left frontal hypoperfusion. Neuropsychological testing revealed normal general cognition as well as bright anterograde memory performances. On retrograde memory tasks, the patient offered a poor performance on Crovitz-Schiffman, with lack of autobiographical memories related to the last 12 months, in spite of a preserved ability to recall public events and to identify public faces. Six months later, after constant and strenuous relearning practice, the patient regained his past and also reported the appearance of some spontaneous recalls. He noticed that the relearned events, compared to the spontaneous recalls, were characterized by a reduced sense of familiarity. Cerebral SPECT was unchanged. A year later he worked as an electrician and denied residual memory problems. No further amnesias emerged during a five year follow-up.

*3.1.2. Patient 2 [76]*

A 30-year-old man with previous rare migraine and doubtful epileptic attacks and a conflictual marriage was admitted on 5th March 1995 after a 'fugue' of 12 hours, followed by an isolated retrograde amnesia covering his whole life with loss of personal identity. General and neurological examination, blood parameters, ECG, EEG, brain CT and SPECT were normal. A search for alcohol and drugs was negative. He appeared alert, cooperative, oriented in time, astonished but not worried. He could not remember anything of his past. The neuropsychological examination revealed preserved general cognition. Memory assessment revealed a selective defect of retrograde memory, disproportionately affecting autobiographical memory, with extremely poor performances on Autobiographical Interview and Crovitz-Schiffman test. Non autobiographical tasks were well performed, apart from a mild defective score on Q 80. Three days later, meeting his relatives, the patient cried and began to regain his memory. On the same day, he was discharged without therapy. One month later, he referred by phone an almost complete recovery. He was then seen by a psychologist who denied the need for psychotherapy. No further neurological or behavioral disturbances have been reported during five years of follow-up. He has changed his job and no further family problems have been noted.

*3.1.3. Patient 3*

This 24-year-old pregnant woman, with a history of epilepsy was admitted unconscious after a seizure on the morning of 15th June 1996. She underwent surgery because of foetal death. On awakening, she exhibited an amnesia for the previous few years, including the fact of being married, pregnant and having another baby. She appeared very worried about being in hospital. When she was told the truth, she had a severe emotional reaction, with depressive symptoms. Self identity was preserved. When she met her husband a few hours later she did not recognize him (she believed she was still married to her first husband). General and neurological examination, blood parameters and ECG were normal. Neuroimaging (brain CT, MRI and SPECT) disclosed a left frontal ischemic lacunar lesion. Neuropsychological examination showed normal general cognition and frailty on verbal learning and recall, which was resolved after 24 hours. A selective retrograde autobiographical amnesia was detected, covering the last five years, while she had no problems on non autobiographical memory, as stated by normal scores on test of Face identification and Familiarity check. After one week the retrograde amnesia was reduced to one year. In the following weeks she reported a gradual shrinkage of the amnesia. She refused to undergo further examinations.

*3.1.4. Patient 4 [76]*

This 49-year-old man had a history of behavior disorders and recurrent episodes of fugue, often beginning with a severe headache. He was admitted on 26th August 1997 because of a sudden loss of consciousness three days after a mild head trauma in the occipital region, after which he had complained of persistent headache. On regaining consciousness, the patient exhibited a complete disappearance of all memories predating the event. General and neurological examinations were normal. Blood and urine parameters, ECG, cerebral CT and SPECT were normal. EEG showed mild bitemporal theta activity. The patient appeared alert, cooperative and disoriented in time and space. A psychiatric consultation noted that the mood was inadequate, with emotive indifference, and repetitive requests to be discharged. He could not give details about his personal identity, his family or his address nor did he remember anything from the past, both personal and public reminiscences being apparently lost. Neuropsychological assessment showed normal general cognition and anterograde memory, documenting a dense retrograde amnesia affecting dramatically both autobiographical and public memory, with a normal performance only in the Face Familiarity check test. Four days after his admission, the patient reported the reappearance of some general memories, mainly concerning his work and his mother, then followed by a gradual memory recovery during several days. He was then seen regularly by the psychiatrist. No other neurological events have been reported since.

*3.1.5. Patient 5 [76]*

This 19-year-old girl had previous episodes of psychogenic dyspnoea. On 19th May 1997, she was found close to her school near her motorbike asking passers-by for help, having completely lost her memory. Admitted, she appeared alert, without physical or neurological deficits, apart from a complete retrograde amnesia, with loss of personal identity. Blood parameters, ECG, EEG and cerebral CT proved normal. When the patient saw her parents a few hours after admission she could not recognize them. Brain SPECT and MRI were normal. Alcohol and drug tests were negative. Neuropsychological examination showed normal general cognition and anterograde memory, documenting a dense retrograde amnesia selectively affecting autobiographical memory. Three days after the acute event, the retrograde amnesia showed a gradual, rapid shrinkage, with a residual mnesic gap covering about one hour. The patient underwent a psychiatric consultation which diagnosed moderate anxiety, but no major psychiatric disorders. The patient was discharged without therapy. No further neurological or neuropsychological problems emerged during three years of follow-up. Anxiety reactions have been subsequently documented in the face of stressful events.

#### *3.1.6. Patient 6*

This 42-year-old man has had a motor disability since age 30, which had been diagnosed as a secondary progressive form of multiple sclerosis (MS), causing severe psychologic distress and recurrent psychotic reactions. He was admitted on 9$^{th}$ December 1998 for an acute ingestion of BDZ resulting in acute respiratory failure and coma lasting three days, then followed by agitation and confusion for one week. CT brain scan proved normal. Neurological examination in January 1999 showed mild pyramidal signs, more pronounced on the right side, and revealed a dense retrograde amnesia. He could not recognize relatives and friends and appeared unaware of having MS. When given this information he developed a depressive reaction and was transferred to the Psychiatric Department. A formal neuropsychological examination in February 1999 showed normal general cognition, with bright performances on anterograde memory tasks, and profound deficits in retrieving past events, selectively affecting ABM. A subsequent cerebral MRI revealed diffuse lucencies. He was then seen regularly by a psychiatrist, and subsequent investigations shed some doubts on the diagnosis of MS, while there emerged a suspect of functional motor disability. No further episodes of amnesia were reported.

#### *3.1.7. Patient 7*

This 16-year-old girl was involved in a car accident on 24$^{th}$ May 1997 resulting in a head injury with global amnesia lasting 24 h associated with vigilance fluctuations, but without focal deficits. No acute brain damage was seen on CT scan, which however revealed a congenital ventricular asymmetry (dx>sn). Once the acute amnesia resolved, she remained unaware of premorbid personal memories, and developed anxiety reactions. Thus, she began to be seen regularly by a psychologist, suspecting a post-traumatic stress disorder. Pretrauma neuropsychiatric history was unremarkable. We saw this girl in February 1999 (aged 18 years) for a neuropsychological consultation. At this time brain MRI was unimpressive. Neuropsychological examination showed frailty on a visuo-spatial attentional task, phonological fluency and phrase construction. Memory performances were bright, except for a selective retrograde amnesia, mainly affecting autobiographical memory, covering most of her life, apart from isolated semantic recalls concerning her time at school. The patient reported also a loss of some procedural knowledge: for example, she had been a dancer since childhood: after the accident, she could no longer remember the dance steps. Personal identity was preserved. We saw the patient regularly until 2001. She suffered from depression and sleep and eating disorders. No further neurologic acute events were reported, but she continued to complain of chronic subjective memory disturbances, with persistence of difficulties in retrieving her past, which she had learned from relatives' reports. We saw the patient again in February 2008, when she reported she had myasthenia gravis. At this time, the memory disorder was unremarkable.

#### *3.1.8. Patient 8*

This 25-year-old woman had a history of recurrent episodes of loss of consciousness during infancy. She reported a psychogenic dyspnoea, maybe related to an episode of sexual assault, for which she has been regularly seen by a psychologist. On 26$^{th}$ June 2000, during a period of strenuous work, she suffered a minor head trauma with a short loss of consciousness (few minutes), after that she exhibited a dense retrograde amnesia. Neurological examination, EEG, brain CT immediately performed proved normal. Psychiatric consultation suggested a mood disorder, mainly focused on anxiety and emotive distress. We saw this girl on 31$^{st}$ July for residual memory problems: she complained of a retrograde amnesia associated with some difficulties in object recognition. Subsequent brain MRI was normal. Cerebral SPECT showed a right frontal hypoperfusion. Formal neuropsychological assessment (2$^{nd}$ August) showed a normal general cognition. Anterograde memory was bright. Retrograde amnesia was deep for ABM, while knowledge of public people, facts and events was slightly involved. The patient was then seen six months later for an episode of loss of consiousness, reasonably a vaso-vagal syncope. At that time, she was neurologically normal and reported a continuous gradual recovery of the retrograde amnesia due to both spontaneous and trained recalls. We saw this girl again in August 2008. She was neurologically normal and reported normal memory and no further neurological or psychological problems. She told us that despite the recovery of retrograde amnesia she still experienced a sense of decreased intimacy for pre-trauma memories.

*3.1.9. Patient 9*

This 39-year-old woman had an uneventual medical history, apart from a previous diagnosis of epilepsy, not on treatment at the time of admission. On 22$^{nd}$ September 2000 she experienced a sudden loss of consciousness lasting few minutes after a heated discussion with her boss. Awakening, she was not able to recognize the place where she worked or her colleagues and exhibited a dense amnesia for the past, including loss of personal identity. Admitted, neurological examination, EEG and brain CT proved normal. She did not recognize her parents, her husband and her seven-year-old daughter. One week later she experienced a sudden reappearance of the first 12 years of life. MRI was normal. Neuropsychological examination (2$^{nd}$ October) showed bright performances on general cognition and anterograde memory. On retrograde memory tasks, she exhibited a deficit of retrieval of personal facts and events, covering most part of her adult life, with poor scores also in the tasks exploring non autobiographical memory. On a subsequent visit on 29$^{th}$ October she reported a further recovery of four years (until age 16). A subsequent cerebral SPECT was normal. She began a trained recovery with a psychologist, with a gradual complete recovery over six months. No further problems were reported during seven years of follow-up.

*3.1.10. Patient 10*

This 19-year-old girl had migraine and previous eating disorders. On the morning of 28$^{th}$ May 2000 she suffered a sudden loss of consciousness during a migrainous attack. On regaining consciousness, she realized she had a retrograde amnesia covering about three years before the attack, including memory for personal events in that period (e.g. her sister's wedding, birth of a niece), and for familiar places and people. EEG and MRI proved normal. Psychiatric interview diagnosed a somatic anxiety. We saw this girl in November 2000 for a neuropsychological consultation. She was neurologically normal. She reported having relearned, not recalled, some autobiographical information which she had previously lost. Neuropsychological examination was normal for general cognition and memory abilities, but still documented a retrograde amnesia, more pronounced for autobiographical events. Cerebral MR-angiography and SPECT proved normal. The amnesia gradually disappeared over the following 6 months. In a telephone interview seven years later, she denied further neurological problems, reporting a normal everyday memory functioning.

*3.1.11. Patient 11*

This 34-year-old woman experienced an acute loss of consciousness on 8$^{th}$ December 2005 while working as usual in her own small dairy on a very cold day. The episode was followed by a profound retrograde amnesia covering 15 years. The next day she experienced a burning headache. She was seen by us five days later, resulting in a normal examination, apart from the retrograde amnesia. EEG and brain CT proved normal. Cerebral PET on 21$^{st}$ December was normal. Psychiatric consultation (December 12) disclosed a personality disorder ("immature and hysteric"), revealing an episode of rape during the patient's childhood, which she had avoided recounting to her parents. Neuropsychological assessment (19$^{th}$ December) gave normal results on general cognition and anterograde memory tasks, apart from reduced digit and spatial span. Autobiographical memory was poor, especially involving the last years of her life. Non autobiographical memory was mildly and partially impaired, with poor scores on "Who is who" and "Dead or alive" tasks. During the subsequent six months, the patient experienced a constant reappearance of old memories, especially those less pleasant. She was regularly seen for psychological counseling. In August 2006 she experienced a day long episode of topographical amnesia, leaving a frailty of geographical orientation. No further episodes occurred during two years of follow-up.

*3.1.12. Patient 12*

This 43-year-old woman with a history of migraine, somnambulism and agoraphobia was admitted for a sudden amnesia during a migraine attack on 14$^{th}$ January 2007. She exhibited normal neurological examination and a retrograde amnesia covering about six years involving autobiographical events. For example she was not aware of having recently lost her mother after a long illness. She did not recognize her house (she had lived there for three years), nor a person she had known since 2001. She was not aware of working in her own shop (she had bought it two years before). Personal identity was preserved. She reported a feeling of being in a very stressful period, which she felt to depend on economic reasons, but without remembering the actual details. The amnesia lasted eight hours, during which EEG and CT brain scan were normal. On 11th October 2007 she was again admitted for a second similar episode. The day before she had had a stressful discussion on work. On the morning after awakening she experienced an intense headache associated with memory disturbances. She took a tablet of a compound including indomethacine and caffeine, with a reduction of headache. Cerebral SPECT performed at the end of the attack was normal. A standard neuropsychological examination was not possible in this patient, given the shortness of both attacks. However, the clinical observation made by one of us (AS) during the attacks suggested a focal FRA, selectively affecting ABM.

#### *3.1.13. Patients 13*

This 38-year-old man had suffered a depression state at age 30 when he lost his father. On 4th December 2008, 24 hours after his father-in-law's funeral, he developed a depressive reaction with crying and anxiety, after which he lost his memory for the past eight years, including home, possessions, friends, neighbours and workplace. Procedural abilities (driving, computing, painting) were preserved. Seen by a psychologist, he was diagnosed with "anxiety disorder with lacunar amnesia". We saw him on 19th December, the neurological examination resulting normal. A neuropsychological assessment on 19th December showed normal general cognition, apart from mild visuo-spatial attentional deficits. Memory performances revealed some difficulties on verbal and spatial learning, and documented a severe retrograde amnesia for both personal and public facts and events, with recollection more impaired than familiarity on non autobiographical tasks. EEG and brain CT proved normal. He was treated with antidepressants. Three months later he reported a reappraisal of some islands of memory. He also reported a brief (20 minute long) episode of spatial disorientation. In June, while working, he experienced a sudden and complete reappraisal of all lost memories. On 30th October 2008, he reported no disturbances, and neurological examination was normal.

Tables 1 and 2 summarize the clinical features and neuropsychological characteristics of the sample. Tables 3 and 4 report details of neuropsychological scores obtained in 12 patients.

## 4. Discussion

All our patients presented an acute event of different nature triggering the onset of an amnesic defect characterized by an impaired recollection of preictal events with preserved or minimally and transiently impaired anterograde memory, thus consistent with a focal retrograde amnesia (FRA). The context favoring onset varied widely: head trauma, migraine attack, 'fugue state', drug overdose/hypoxia, seizure, syncope, acute emotive reaction. No clear triggering factors were elicited in one patient (case 5), even though she may have been involved in a minor car accident. All but two patients had suffered from neurological or psychological problems, usually not so consistent as to interfere with their lives. In general, the ancillary investigations (general, neurologic, neuroradiologic and neurophysiologic) performed during the amnesic phase were not consistent with overt brain damage. The only neuroimaging abnormalities were a selective frontal SPECT hypoperfusion in three patients (left in two). Psychiatric interview disclosed minor mood/affective disturbances in ten. The retrograde amnesia was profound in most cases, always affecting autobiographical memory, with a defect often so dense as to cover the whole of patients' lives (seven cases) and to erase the knowledge of their own identity (four cases). Semantic and episodic non autobiographical memories were variably affected, being spared in seven patients. Procedural memory was spared in all but one patient (patient 6). This girl reported the loss of some procedural abilities, in particular dancing, but the accident she experienced also caused temporary orthopedic problems, so it is difficult to establish exactly the nature of her difficulty to return to dancing. Otherwise, it is also to be taken into account that she was the only patient with a delayed and maybe incomplete recovery of FRA.

The outcome of FRA was favourable, the amnesic defect being reversed or improved in all, with two outcomes: rapid shrinkage within a few days, or more gradually during weeks or months (max. six), leaving a permanent gap for the onset. The recovery time was prolonged (two years) and incomplete in one patient. No consistent sex predominance was evident. Age at occurrence spanned adolescence and young adulthood, whereas we did not encounter cases in middle-old age.

Some issues merit detailed comment. Firstly, some of these patients (pts 2, 4, 5, 11, 13) presented conditions often considered suggestive for a psychogenic nature [3,29,35,49,51,69]: background of conflictual conditions, wandering (in case 2 a true 'fugue' through remarkable distances), asking for help in a non medical facility, loss of personal identity, transience and short duration of RA, prominent autobiographical impairment, recovery on meeting relatives. On the other

Table 1
Clinical and neuroradiological characteristics of the sample

| Pt | Age | Sex | Neuropsychiatric antecedents | Associated/trigger events | Neuroimaging | Psychiatric interview |
|---|---|---|---|---|---|---|
| 1 | 20 | M | - | mild head trauma | CT/MRI: normal<br>SPET: left frontal hypoperfusion | normal |
| 2 | 30 | M | epilepsy ?, migraine | fugue, stress | CT/MRI/SPET: normal | normal |
| 3 | 24 | F | epilepsy | pregnancy, seizure | CT/MRI: left frontal ischemic lacuna<br>SPET: left frontal hypoperfusion | situational depression |
| 4 | 49 | M | behavior disorders, migraine | posttraumatic headache<br>loss of consciousness | CT/SPET: normal | emotive indifference |
| 5 | 19 | F | psychogenic dyspnoea | road accident ? | CT/MRI/SPET: normal | anxiety |
| 6 | 42 | M | motor impairment (multiple sclerosis ?) | BDZ overdose, cerebral hypoxia | CT: normal<br>MRI: diffuse lucencies | depression |
| 7 | 16 | F | – | head injury | CT/MRI: congenital ventricular asymmetry | posttraumatic stress disorder |
| 8 | 25 | F | psychogenic dyspnoea, | mild head trauma | CT/MRI: normal<br>SPET: right frontal hypoperfusion | anxiety, depression |
| 9 | 39 | F | epilepsy | loss of consciousness (seizure?) | CT/MRI/SPET: normal | normal |
| 10 | 19 | F | eating and affective disorders, migraine | migraine attack, loss of consiousness | CT/angioMRI/SPET: normal | somatic anxiety |
| 11 | 34 | F | child abuse, migraine | headache, loss of consciousness | CT/PET: normal | personality disorder |
| 12 | 43 | F | somnambulism, migraine, agoraphobia | migraine attack | CT/SPET: normal | anxiety |
| 13 | 38 | M | depression | sad event | CT: normal | anxiety |

Table 2
Results of the clinical and neuropsychological assessment and follow-up

| Pt | Personal identity | General cognition | Anterograde amnesia | Autobiographical RA | Non autobiographical RA | Follow-up |
|---|---|---|---|---|---|---|
| 1 | spared | normal | transient | yes (1 yr) | no | full recovery (6 months) |
| 2 | lost | normal | no | yes (all life) | no | full recovery (3 days) |
| 3 | spared | normal | transient | yes (5 yrs) | no | full recovery (2 weeks) |
| 4 | lost | normal | no | yes (all life) | yes | full recovery (2 weeks ) |
| 5 | lost | normal | no | yes (all life) | no | full recovery (3 days) |
| 6 | spared | normal | no | yes (all life) | no | full recovery (6 weeks) |
| 7 | spared | attentional and executive frailty | transient | yes (all life) | partial | improvement |
| 8 | spared | normal | no | yes (all life) | partial | full recovery (6 months) |
| 9 | lost | normal | no | yes (all life) | yes | full recovery (6 months) |
| 10 | spared | normal | transient | yes (3 yrs) | partial | full recovery (6 months) |
| 11 | spared | reduced digit and visuo-spatial span | no | yes (15 yrs) | partial | full recovery (6 months)<br>1 TTA episode |
| 12 | spared | normal* | no* | yes (6 yrs)* | no* | full recovery (8 hours)<br>relapse after 9 months |
| 13 | spared | mild attentional and visuo-spatial deficits | no | yes (8 yrs) | yes | full recovery (6 months)<br>1 TTA episode |

RA = retrograde amnesia; TTA = transient topographical amnesia.
*Note: Patient 12 was not formally tested, results were inferred by the clinical interview.

hand, predisposing factors for global psychogenic amnesia [37], such as previous major psychiatric disorders or transient amnesic dysfunction, and severe precipitating stress were occasionally present and there was a high prevalence of true organic events triggering FRA (head injury, migraine, seizures). This raises difficulties in labeling our cases as psychogenic at all and contrasting features were seen. Loss of personal identity,

Table 3
Neuropsychological scores obtained on tasks of general cognition and anterograde memory

| | Range | pt. 1 CS (ES) | pt. 2 CS (ES) | pt. 3 CS (ES) | pt. 4 CS (ES) | pt. 5 CS (ES) | pt. 6 CS (ES) | pt. 7 CS (ES) | pt. 8 CS (ES) | pt. 9 CS (ES) | pt. 10 CS (ES) | pt. 11 CS (ES) | pt. 13 CS (ES) | Cut-off |
|---|---|---|---|---|---|---|---|---|---|---|---|---|---|---|
| *General Cognition* | | | | | | | | | | | | | | |
| Attentional Matrices [71] | 0–60 | 34.2 (1) | 60 (4) | NA | 49.2 (4) | 47.7 (3) | 32.25 (1) | 33.75 (1) | 36 (1) | 60 (4) | NA | 44.75 (3) | 31.25 (1) | 31 |
| Word Fluency (F, A, S) [7] | | 39 (4) | 31 (4) | NA | 26.3 (3) | 33.3 (4) | 44.7 (4) | 14.4 (0) | 53 (4) | 44 (4) | NA | 32.3 (4) | 10.7 (0) | 17.3 |
| Phrase Construction [7] | 0–25 | 25 (4) | 13.3 (1) | NA | 18 (2) | 19.2 (3) | 22.2 (4) | 11.5 (1) | 25 (4) | NA | NA | 25 (4) | 14.7 (2) | 8.7 |
| Copy of Rey's Figure [2] | 0–36 | 36 | 36 | 36 | 35 | 36 | 36 | 33.56 | 36 | 36 | 36 | 33 | 36 | 28 |
| Wisconsin Card Sorting [55] | 0–6 | NA | 6 | NA | 4 | 6 | NA | 6 | 6 | 6 | NA | 6 | NA | 2 |
| Raven's Matrices (1947) [7] | 0–36 | 22.5 (2) | 30.5 (4) | 29.6 (4) | 27.2 (3) | 29.2 (4) | 34.3 (4) | 24.8 (2) | 28.9 (4) | 28.8 (4) | 28.2 (4) | 30.9 (4) | 27.5 (3) | 18.9 |
| *Anterograde memory* | | | | | | | | | | | | | | |
| Digit Span [59] | 0–9 | 6 (4) | 5.75 (4) | 5.75 (4) | NA | 6.5 (4) | 7 (4) | 4.75 (3) | 7 (4) | 6.5 (4) | 7.5 (4) | 3 (0) | 3 (0) | 3.7 |
| Verbal Span [71] | 0–10 | 5.25 (4) | 4.5 (4) | 4.25 (3) | 4.7 (4) | 3.2 (1) | NA | 3.5 (2) | NA | NA | NA | 3.5 (2) | NA | 3 |
| Corsi's Blocks [59] | 0–9 | 5.5 (4) | 4.5 (3) | 3.75 (1) | 4.5 (3) | 4.75 (4) | 4.5 (3) | 4.75 (4) | 6.75 (4) | 5.75 (4) | 6.25 (4) | 3.75 (1) | 3.5 (1) | 3.7 |
| Immediate Visual Memory [7] | 0–22 | 22 | 19.3 (3) | NA | 21 (4) | 22 (4) | 19.8 (4) | 18.2 (2) | NA | 22 (4) | 19.1 (3) | 17.6 (2) | 14.8 (1) | 13.8 |
| Rey's 15 Words[7]: Short-term | 0–75 | NA | NA | NA | 42.3 (4) | NA | 42.5 (4) | 47.9 (4) | 46.9 (4) | 41.8 (4) | NA | 43.2 (4) | 39.5 (3) | 28.5 |
| Rey's 15 Words: Long-term | 0–15 | NA | NA | NA | 7.2 (3) | NA | 11.7 (4) | 9.2 (4) | 9.2 (4) | 12.1 (4) | NA | 10.3 (4) | 7.7 (3) | 4.6 |
| Verbal learning [71]: total recall | 0–180 | 106 (3) | 116 (4) | 80 (2) | 49 (1) | 108 (3) | NA | NA | 100 (3) | NA | 102 (3) | 118 (4) | NA | 37 |
| Verbal learning: non-casual recall | 0–180 | 96 (4) | 107 (4) | 0 (0) | 9 (2) | 96 (4) | NA | NA | 69 (4) | NA | 89 (4) | 115 (4) | NA | 2 |
| Verbal learning: delayed recall | 0–10 | 3.75 (1) | 10 (4) | 3.75 (1) | 3.2 (1) | 10 (4) | NA | NA | 10 (4) | NA | 10 (4) | 10 (4) | NA | 2.25 |
| Paired Associate Learning [57] | 0–22.5 | NA | 17 (4) | NA | 8.5 (2) | 12 (4) | 18 (4) | 20 (4) | 17.5 (4) | 22.5 (4) | NA | 15.5 (4) | 2.5 (0) | 6.5 |
| Story Recall [71] | 0–16 | 9.75 (2) | 10.6 (2) | 10.65 (2) | 6.9 (1) | 14 (4) | 14.9 (4) | 16 (4) | 12.65 (4) | 12.55 (3) | NA | 14 (4) | 5 (1) | 4.7 |
| Supra-span Spatial Learning [71] | 0–29 | 29 (4) | 27.1 (4) | 0 (0) | 11 (2) | 14 (3) | 16.05 (3) | 15.79 (3) | 15.49 (3) | 10.39 (2) | 19.21 (4) | 11.33 (2) | 2.45 (0) | 5.7 |
| Rey's Figure Recall [2] | 0–36 | 24 | 13.8 | 13.03 | 21.3 | 15.4 | 12.5 | 16.51 | 19 | 12.1 | 8.07 | 12.62 | 28.06 | 6.2 |

CS = corrected score; ES = equivalent scores [see 71 for details]; NA = not administered. (Note: Patient 12 was not formally tested)

Table 4
Neuropsychological scores obtained on retrograde memory tasks

| | Range | pt 1 CS | pt 2 CS | pt 3 CS | pt 4 CS | pt 5 CS | pt 6 CS | pt 7 CS | pt 8 CS | pt 9 CS | pt 10 CS | pt 11 CS | pt 13 CS | Cut-off |
|---|---|---|---|---|---|---|---|---|---|---|---|---|---|---|
| *Non autobiographical* | | | | | | | | | | | | | | |
| Famous Events Questionnaire [12] | 0-80 | 62.5 | 25.7 | NA | NA | NA | 45.63 | NA | NA | NA | NA | NA | NA | 32.4 |
| Face Identification (errors) [18] | 32-0 | 0.75 | 1.87 | 0.35 | 6.9 | 0 | 0.91 | 2.95 | 2.42 | 4.09 | 1.87 | 0.37 | 0 | 2.99 |
| Face Familiarity check (errors) [18] | 36-0 | 0.76 | 1.93 | 0 | 1.99 | 0.48 | 0.22 | 2.53 | 3.07 | 4.07 | 2.32 | 0.11 | 0.91 | 2.05 |
| Famous Names Familiarity check | 0-21 | NA | 19 | NA | 0 | 19 | 17 | 18 | 14 | 4 | 20 | NA | NA | 20 |
| Current Famous Events | 0-11 | 11 | 7 | NA | 0 | 9 | 7 | 2 | 5 | 0 | 3 | 6 | 4 | 6 |
| "Who is who" | 0-23 | NA | 22 | NA | 0 | 21 | 17 | 17 | 13 | 4 | 11 | 14 | 9 | 22 |
| "Dead or alive" | 0-27 | NA | 19 | NA | NA | 25 | NA | NA | 1 | 8 | 16 | 11 | 8 | 16 |
| *Autobiographical* | | | | | | | | | | | | | | |
| Autobiographical Interview [4] | | NA | | | | | | | | | NA | | | |
| Scholar age | 0–15 | – | 2 | 10 | 1 | 2 | 2 | 3 | 0 | 5 | – | 15 | 15 | |
| Adolescence | 0–15 | – | 0 | 2 | 0 | 3 | 0 | 0 | 0 | 5 | – | 15 | 8.5 | |
| Last two years | 0–15 | – | 0 | 0 | 0 | 0 | 0 | 0 | 0 | 0 | – | 0 | 0 | |
| Global score | 0–45 | – | 2 | 12 | 1 | 5 | 2 | 3 | 0 | 10 | – | 30 | 23.5 | 19.5 |
| Crovitz-Schiffman [21] | 0–180 | 120 | 12 | NA | 5 | 63 | 39 | 74 | 0 | 78 | 73 | 77 | 75 | 125 |

CS = corrected score; NA = not administered. (Note: Patient 12 was not formally tested.)

Table 5
Classification of FRA

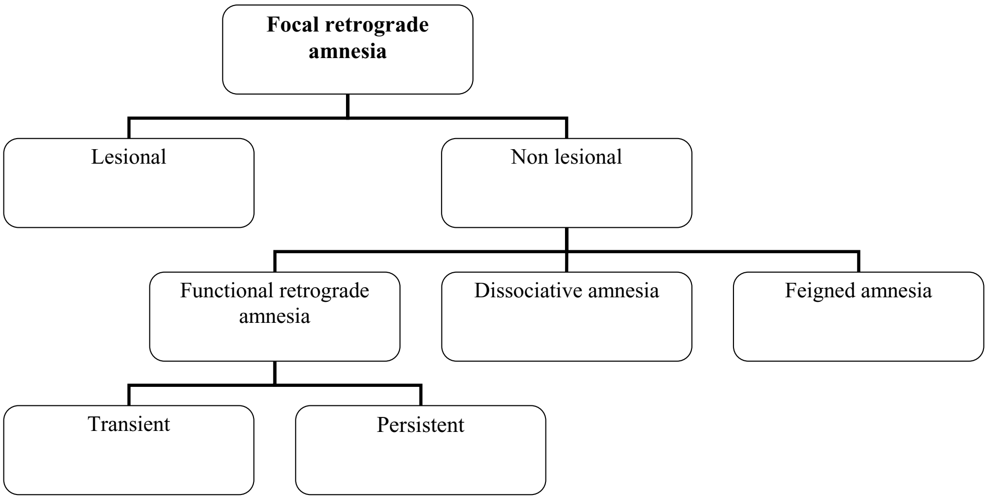

often invoked as a marker of psychogenic amnesia, was present in a patient who exhibited FRA after a seizure (pt. 3). Malingering was also considered, but no aspects favouring such hypothesis emerged in any patient, in particular lacking clear evidence of consistent external incentives. Thus we believe that all our patients can be seen as typical examples of "functional" FRA according to De Renzi and coworkers [13–15] and recently reappraised [20,76]. An analysis of our patients' characteristics led us to pinpoint a number of features applicable to most of them, suggesting they represent possible cardinal features for functional FRA:

1. History of neurological or minor psychiatric disorders
2. Triggering factors for the acute amnesia
3. Dense impairment of ABM
4. Lack of focal neurological signs or symptoms
5. No evidence of cerebral lesions on neuroimaging
6. Favourable outcome
7. No or low recurrence rate.

Point 6 deserves some comment. Recovery occurred in two different ways: sudden, early reappraisal of spontaneous memories or more gradual and tardive relearning of lost memories, usually lasting several months, in one case years. The result is the complete reconstitution of the patient's history, but with a different sense of veridicity. So, all the patients recovered, in the sense of knowledge of personal past events, but reappraisal was only spontaneous for some, including the sense of living memories. Obviously the two outcomes should be considered different, and both were present in previous cases. The early sudden reappearance of lost memories has been well described as the "Petit Madeleines" phenomenon [43] and examples of the second type of recovery have been reported [65, 66]. Other aspects, such as onset with wandering, loss of personal identity, defective memory for non personal facts or events, were seen in only some of the cases, suggesting they are adjunctive or optional criteria, present occasionally but not contrasting the diagnosis of FRA.

A second intriguing issue is the constant impairment of autobiographical memory (ABM). We previously hypothesized [76] that ABM may be more vulnerable to stressful events, especially in subjects with predisposing psychosocial factors and emotionally highly involved, due to the particular organization of ABM. According to the recently reported model of Conway and Pleydell-Pearce [10], there are two forms of ABM retrieval: direct and generative. The main difference between the two types of retrieval is that the search process is modulated by control processes in generative retrieval but not, or less, in direct retrieval. In the case of direct retrieval it is postulated that a cue maps directly onto a very specific episode-like representation in long-term memory and this then automatically comes to mind. However in the case of normal retrieval such a representation could not enter consciousness unless it was linked into more abstract autobiographical knowledge which contextualises it, probably under executive control processes. The prominent autobiographical amnesic defect of FRA patients may be explained by a lost access to abstract autobiographical knowledge, due to a temporary executive control dysfunction, possibly favoured by a frontal disengagement [9,10]. When this occurs, even though patients are able to recall specific memories, they have no abstract context in which to place those memories: these are probably instances of direct retrieval, but because access to more abstract contextualising autobiographical memory is not possible, these memories are relatively meaningless to those recalling them [76].

The third point is the meaning of the SPECT frontal hypoperfusion found in three patients. We previously hypothesized a retrieval deficit associated with frontal lobe temporary disengagement to explain functional FRA [76]. The neural correlates of FRA remain uncertain, but hippocampi/medial temporal lobes and frontotemporal neuronal networks are known to be involved in the process of retrieving old memories and autobiographical events [8,19,25,41]. A recent review of neuroimaging findings emphasized the role of the prefrontal cortex in the retrieval of episodic memories [22]. In particular, the right mid-dorsolateral prefrontal cortex seems to support the retrieval of non-personal episodic memory, whereas ventromedial prefrontal regions, predominantly on the left side, are associated with the retrieval of autobiographical events. A recent case of FRA fugue associated with migraine showed frontal hypoperfusion during the amnesic phase [62] and similarly these structures seem impaired when a psychogenic mechanism is involved [26]. Thus, even though our finding of frontal hypoperfusion in three patients precludes any conclusions, it is consistent with previous observations implicating the frontal region in the genesis of FRA.

Apart from the above speculative comments, our experience confirms previous suggestions that FRA represents a syndrome, potentially triggered by different etiologies [30,44]. The FRA syndrome clearly comprises a non lesional – more common in our experience – "functional" form, known as a mnestic block [51,52], in which evidence of a psychiatric background is the rule [20].

Something similar occurs in transient global amnesia (TGA), a more common and better known temporary amnestic syndrome. Although TGA and functional FRA occur at different ages (functional FRA in young people, TGA in middle-old age), they share several features. A sudden block of memory occurs in both conditions following either a cerebral insult like concussion, seizures, intoxication, etc. or an emotional shock, with a relatively uniform pattern in all patients in the absence of proven cerebral lesions. Both are transient, albeit with different duration (a few hours for TGA, hours to months for functional FRA), associated with possible residual memory frailty. The pathomechanisms involved in TGA are still poorly understood. Current hypotheses favor temporary perturbation of hippocampal functioning more than structural damage, namely Leao's spreading depression [24,58]. This implies that that stress, emotional and/or physical experiences may induce biochemical changes in the brain, triggering a spreading depression, leading to a decreased local metabolism and a reversible functional ablation of the structures critical for memory. Spreading depression, propagating in different parts of the brain, may induce varied types of amnesia depending on the affected region and the duration and density of the phenomenon. Psychological and emotional instability are commonly found in TGA series, suggesting that they may be particularly sensitive to psychological stress [63]. Stressful events, as well as drugs and trivial head trauma, have been reported to elicit TGA in young people [63]. We hypothesize that something similar may occur in FRA, particularly in the functional form. Thus, we disagree with Kritchevsky et al. [40] who suggested an organic origin for TGA based on the uniformity of the attack, and a psychogenic nature for FRA given the heterogeneity of its presentation. Data from our patients indicate that FRA, especially in its functional form, has salient uniform features with minor differences in the characteristics of amnesia presentation, probably depending on the severity of the memory block. The same occurs for the so-called partial forms of TGA, reported with increasing frequency in recent years [27,33,56,72,74,75] and initially construed as psychogenic events. Transient short-lasting cases of FRA have also been described ([60,77], and case 12 of the present sample) and two patients of the present sample (11 and 13) experienced a subsequent episode of transient topographical amnesia, considered a partial form of TGA. In addition, one of the first reported cases of FRA was related to a TGA episode [67], reinforcing the idea that these events are triggered by similar mechanisms.

In conclusion, our observations strengthen the assumption that FRA is a syndrome, triggered by physical or mental trauma, and confirm the existence of a functional form, not necessarily psychogenic in nature, which merits the dignity of a true neurological condition, as for TGA. A tentative nosographic classification of FRA is given in Table 5 and includes: lesional and non lesional forms, the latter comprising functional retrograde amnesia (as stated by De Renzi [15]), genuine psychogenic amnesia (dissociative amnesia) and simulated cases, as stated by DSM IV [16]. Functional FRA includes a transient form and a more persistent form [14,26,66,67]. It is still unclear whether the transient and persistent forms are different diseases or different severities of the same disease, but collecting and studying wider samples of patients will better define the taxonomy of FRA.
